# Investigation of Microstructure and Mechanical Performance of IN738LC Superalloy Thin Wall Produced by Pulsed Plasma Arc Additive Manufacturing

**DOI:** 10.3390/ma13183924

**Published:** 2020-09-04

**Authors:** Kaibo Wang, Zhe Sun, Yuxin Liu, Yaohui Lv

**Affiliations:** National Key Laboratory for Remanufacturing, Academy of Army Armored Force, Beijing 100072, China; wkb03632@alumni.sjtu.edu.cn (K.W.); zhesun.rm@foxmail.com (Z.S.)

**Keywords:** microstructure, mechanical performance, IN738LC, pulsed plasma arc additive manufacturing, cracking mechanism

## Abstract

The IN738LC Ni-based superalloy strengthened by the coherent γ′-Ni_3_(Al,Ti) intermetallic compound is one of the most employed blade materials in gas turbine engines and IN738LC thin wall components without macro-cracks were fabricated by pulsed plasma arc additive manufacturing (PPAAM), which is more competitive when considering convenience and cost in comparison with other high-energy beam additive manufacturing technologies. The as-fabricated sample exhibited epitaxial growth columnar dendrites along the building direction with discrepant secondary arm spacing due to heat accumulation. A lot of fine γ′ particles with an average size of 81 nm and MC carbides were observed in the interdendritic region. Elemental segregation and γ–γ′ eutectic reaction were analyzed in detail and some MC carbides were confirmed in the reaction L + MC→γ + γ′. After standard heat treatment, bimodal distribution of γ′ phases, including coarse γ′ particles (385 nm, 42 vol.%) and fine γ′ particles (42 nm, 25 vol.%), was observed. The mechanism of microstructural evolution, phase formation, as well as cracking mechanisms were discussed. Microhardness and tensile tests were carried out to investigate the mechanical performance. The results show that both the as-fabricated and heat-treated samples exhibited a higher tensile strength but a slightly lower ductility compared with cast parts.

## 1. Introduction

Nowadays, additive manufacturing (AM) is a promising technology to repair and fabricate functional metallic materials. AM is able to manufacture complex components by virtue of stacking material layer by layer, reducing the machining process over conventional or wrought parts, such as turbine discs and blades [1,2,3,4]. Pulsed plasma arc additive manufacturing (PPAAM) combining pulsed plasma arc welding with AM is a promising technique capable of forming full-density near-net-shape components and is competitive in terms of its productivity and cost compared with laser and electron beam additive manufacturing [5,6]. In addition, a plasma arc always possesses a higher energy density, better stability and is more accessible for the precise control of heat input in comparison with conventional electric arcs. Thus, PPAAM is particularly appropriate for fabricating aircraft engine parts with complex geometry. It is reported that Inconel 625, Inconel 718, and Ti-6Al-4V thin wall components have been successfully built through PPAAM [7,8,9].

IN738LC Ni-based superalloy, strengthened by the ordered, coherent γ′-Ni_3_(Al,Ti) precipitates, is one of the most employed blade materials and has been widely used in land-based and aero gas turbine engines. The IN738LC superalloy has a very excellent high-temperature strength, creep rupture strength and hot corrosion resistance and thus can work at elevated temperatures up to 980 °C [10]. Various alloying elements in the IN738LC play an important role in the strengthening. Cr, Co, W and Co can achieve effective reinforcement of the γ-solid solution through enhancing the lattice distortion and thus increasing slip resistance [11]. Nb, Ti, and Ta can replace some Al and increase the lattice mismatch of γ′. It is known that the internal stress field around the γ′ phase can be enhanced remarkably with the lattice mismatch between γ and γ′ increasing, while the stability of γ′ is weakened [12]. IN738LC contains large amounts of Al and Ti (>6%, wt.%), which make it difficult to weld because of its high crack susceptibility. Solidification cracks, liquation cracking, and strain age cracking have been reported in the fusion zone or heat-affected zone (HAZ) during welding [13,14,15]. AM has been used to fabricate IN738LC components with minimal or even no presence of hot cracks [16,17,18]. 

Several laser additive manufacturing technologies have been recently used to form IN738LC components, such as laser consolidated (LC) [19], laser solid forming (LSF) [20], laser metal deposition (LMD) [17], and selective laser melting (SLM) [21]. Although these processes use similar energy sources, their process configuration and parameters are unique. Consequently, the as-fabricated IN738LC samples show different microstructural characteristics and mechanical performance. Compared with cast ingots, the as-fabricated samples possess finer grains and slight microsegregation. The microstructure of the as-fabricated samples mainly consists of MC carbides, γ′ phases, and eutectic γ–γ′ constituents. M_23_C_6_ carbides, M_2_B borides, and Ni-Zr intermetallic compounds are found in grain boundaries and the interdendritic region [17,22]. However, γ′ phases are not necessarily observed in the as-fabricated sample due to the high cooling rate, which can suppress any precipitation of the γ′ phase [19]. Therefore, heat treatment is always known as an effective procedure to adjust the microstructure and enhance the corresponding mechanical properties.

Almost all the previous research about AM of IN738LC focuses on laser additive manufacturing technologies [17,19,20]. In the present study, PPAAM was carried out to form an IN738LC thin wall and the heat input decreased gradually to stabilize the molten pool and reduce the heat accumulation. The microstructure and mechanical properties of the as-fabricated sample were investigated in detail, and the cracking mechanism was also illustrated. In addition, the effect of post-heat treatment on the microstructure and mechanical performance of the as-fabricated IN738LC component was studied as well.

## 2. Experimental Procedure

### 2.1. Equipment

**Figure 1:**
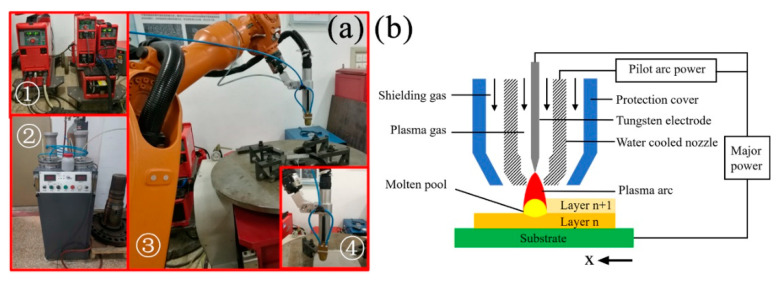
Pulsed plasma arc manufacturing: (**a**) the system of pulsed plasma arc additive manufacturing (PPAAM) and (**b**) the principles of PPAAM process; ① plasma arc power source; ② powder feeder; ③ six-axis robot; ④ coaxial powder-feeding torch.

As shown in Figure 1a, the system of PPAAM consists of a plasma arc power source (Fronius Magic Wave 3000, major power and Plasma Module 10, pilot arc power, Austria), a plasma welding torch (Castolin E 42, coaxial powder feeding, Germany), a powder feeder (DPSF-2, China), and a six-axis robot (KUKA KR6, Germany), which is used to control the motion accuracy of the welding torch. The inert gas Ar is used not only as a plasma gas and a shielding gas, but also as a medium to transport IN738LC powder in the powder feeder. Figure 1b presents the principles of the PPAAM process. The plasma arc in the present study is a kind of transferred arc, which is generated through the cooperation between pilot arc power and major power. During the PPAAM process, IN738LC powder was fed into the molten pool heated by the pulsed plasma arc and solidified rapidly onto the previously formed layer.

### 2.2. Materials and Sample Manufacturing

**Figure 2:**
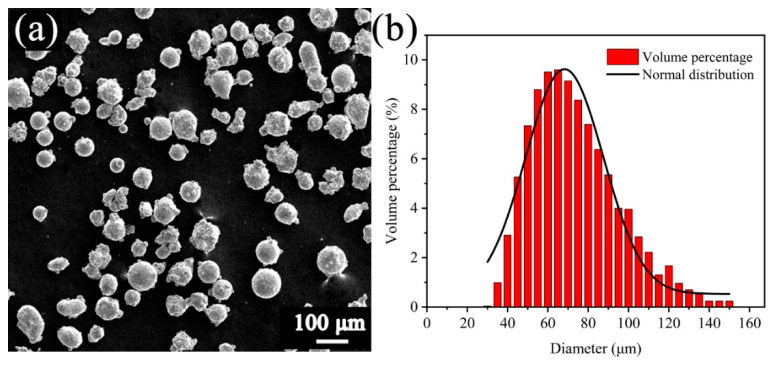
The as-received IN738LC powder: (**a**) the morphology and (**b**) the size distribution.

Commercial IN738LC powder (Carpenter Powder Products, USA) with an average size of 71 μm was used in the present study. Figure 2 shows the morphology and size distribution of the as-received IN738LC powder. Most of the powder particles demonstrate a spherical shape with a few smaller satellite particles on the surface. Ninety percent of these powder particles range from 46 μm to 102 μm. The chemical composition of IN738LC powder is shown in Table 1.

**Figure 3:**
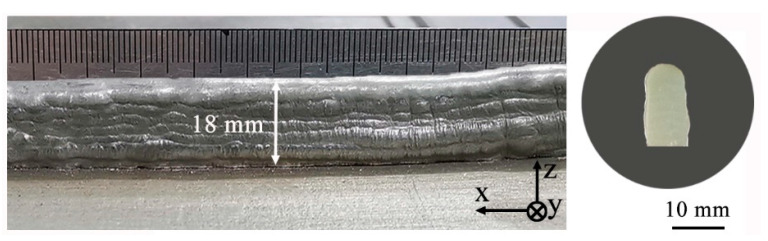
The IN738LC thin wall fabricated by PPAAM.

During the PPAAM of IN738LC thin wall components, the depositing direction was kept constant along the positive X-axis, while the building direction was kept positive along the Z-axis, and the Z-increment between layers was maintained at 1.5 mm. After 12 layers were deposited, a IN738LC thin wall free of macrocracks with dimensions of 100 mm × 8 mm × 18 mm (thickness of 8 mm) was successfully fabricated, as can be seen in Figure 3. It is worth noting that, in order to reduce heat accumulation, the peak current decreased gradually, and the average power can be calculated according to the literature [23], as listed in Table 2, while some other parameters were kept constant: the ratio of the base current to the peak current was 60%, the duty cycle was 50%, the pulse frequency was 15 Hz, the scanning speed was 0.1 m/min, the powder feeding rate was 10 g/min, the plasma gas flow was 0.2 L/min, and the shielding gas flow was 15 L/min. To enhance the performance of the as-fabricated IN738LC sample, post-heat treatment (1120 °C, 2 h/air cooling + 850 °C, 24 h/air cooling) was carried out in a muffle furnace with an argon atmosphere.

### 2.3. Microstructural Characterization and Mechanical Testing

Samples were cut on the transverse sections (y-z plane) along the build direction by electrical discharge machining (EDM) for microstructural characterization and XRD examinations. Specimens used for Olympus optical microscope (OM) observation were etched for 15–30 s with a solution of 100 mL C_2_H_5_OH + 20 mL HCl + 5 g FeCl_3_, while electrolytic etching in a solution of 12 mL H_3_PO_4_ + 40 mL HNO_3_ + 48 mL H_2_SO_4_ at 6 V for 4–6 s was used for FEI NOVA NANOSEM scanning electron microscope (SEM) observation, equipped with an energy-dispersive X-ray spectrometer (EDS) and an Oxford electron backscattered diffraction (EBSD). XRD was performed by using Cu Kα radiation (λ = 0.15425 nm) and the diffraction profile ranged from 25° to 105° with an angle step of 0.01°. Microstructural examination was also carried out with a TECNAL G2 transmission electron microscope (TEM). The size and volume fraction of the γ′ particles were acquired by ImageJ software. The microhardness of samples was measured with an HXZ-1000 micro Vickers hardness tester and the load was set to 200 g with a dwell time of 15 s. Tensile samples were machined according to our previous work [9]. Tensile tests, according to the ASTM-E8 standard, were carried out on an AG-25KNIS machine with an extension rate of 0.2 mm/min.

## 3. Results and Discussion

### 3.1. Grain Structures and Growth in PPAAM of IN738LC

**Figure 4:**
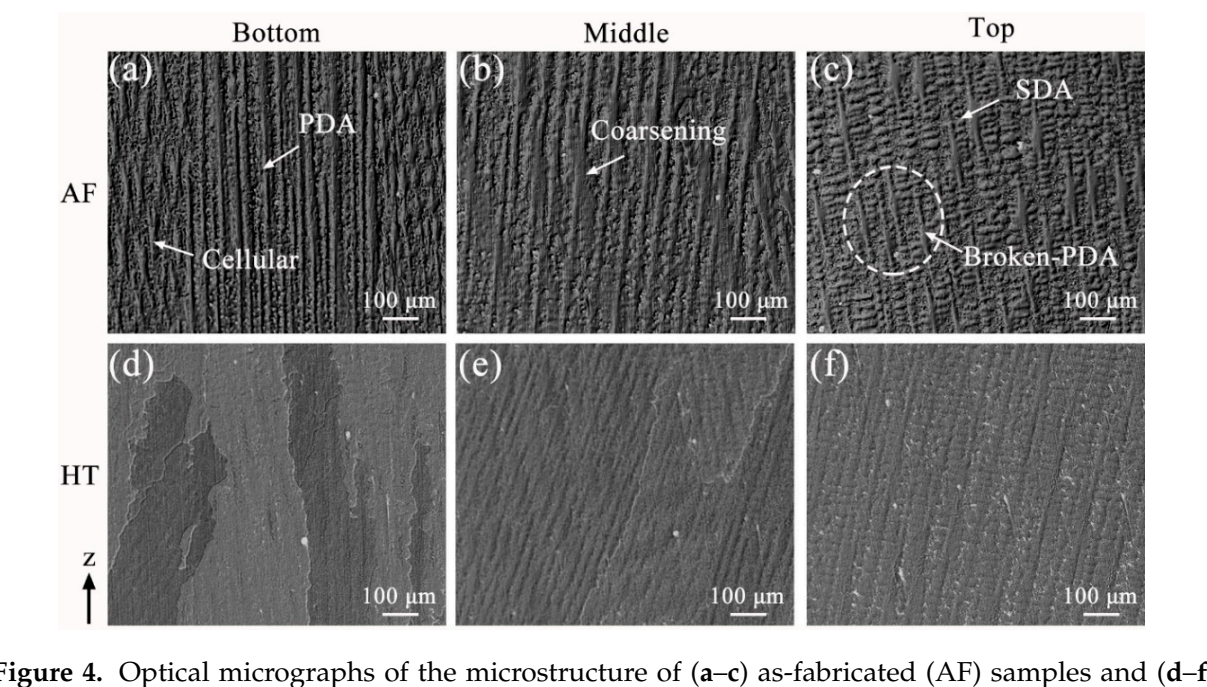
Optical micrographs of the microstructure of (**a**–**c**) as-fabricated (AF) samples and (**d**–**f**) heat-treated (HT) samples.

Optical micrographs of the as-fabricated (AF) samples and heat-treated (HT) samples are shown in Figure 4. Abundant fine columnar dendrites, resulting from rapid solidification in PPAAM, can be observed in the AF samples. Due to heat accumulation during fabrication, different locations of the AF sample contain discrepant grain morphologies. Figure 4a–c present the microstructure in the bottom, middle and top region of the AF sample, respectively. The dendrite arm spacing was measured according to the literature [24]. In the bottom region, many fine columnar dendrites with some cellular dendrites aligning at the sides can be found. Very tiny secondary dendrite arms (SDA), perpendicular to the primary dendrite arms (PDA), demonstrate an immature growth and the average SDA spacing is found to be 12.1 μm. Coarse PDA with a few SDA appears in the middle region and the average SDA spacing increases to 16.2 μm. When it comes to the top region, SDA grow more mature with an average spacing value of 21.5 μm. Some SDA experience re-melting and break away from the PDA, leading to the occurrence of broken PDA, which could be attributed to the solute driving mechanism in the research of Dutta and Rettenmayr [25]. Various morphologies of grains growing in the samples produced via the AM process are typically decided by the temperature gradient (G) and growth rate (R). Kurz and Fisher proposed an interface morphology parameter (G/R) [26]. As it decreases, the grain morphology could change from columnar dendrites to cellular dendrites, and to equiaxial dendrites due to the constitutional undercooling. It is noted that heat accumulation during PPAAM could contribute to the reduction in G/R because of the decreasing G, which is also related to the cooling rate. The SDA spacing has been reported to be dependent on the cooling rate and follow an equation of the type [27]:

(1) $$\lambda =k_{c}\varepsilon^{n}$$

where *λ* is the SDA spacing, *ε* is the cooling rate, and *k_c_* and *n* is the constant. Using the values of *k_c_* = 518.39 and *n* = −0.592, as determined by other researchers for Ni-based superalloys [28], the cooling rate in the bottom, middle and top sections of AF samples fabricated by PPAAM are calculated to be 571 K/s, 349 K/s and 216 K/s, respectively. These are more considerable values compared to those in the conventional casting process, which are normally less than 5 K/s. After heat treatment, the grain morphologies change a little, as shown in Figure 4d–f, corresponding to the bottom, middle and top region in the HT samples. It is known that elemental segregation in the interdendritic region could be weakened or eliminated during heat treatment. The difference in elemental distribution in the interdendritic region and dendritic core is inferred to be weakened due to the poor visibility of dendritic boundaries.

**Figure 5:**
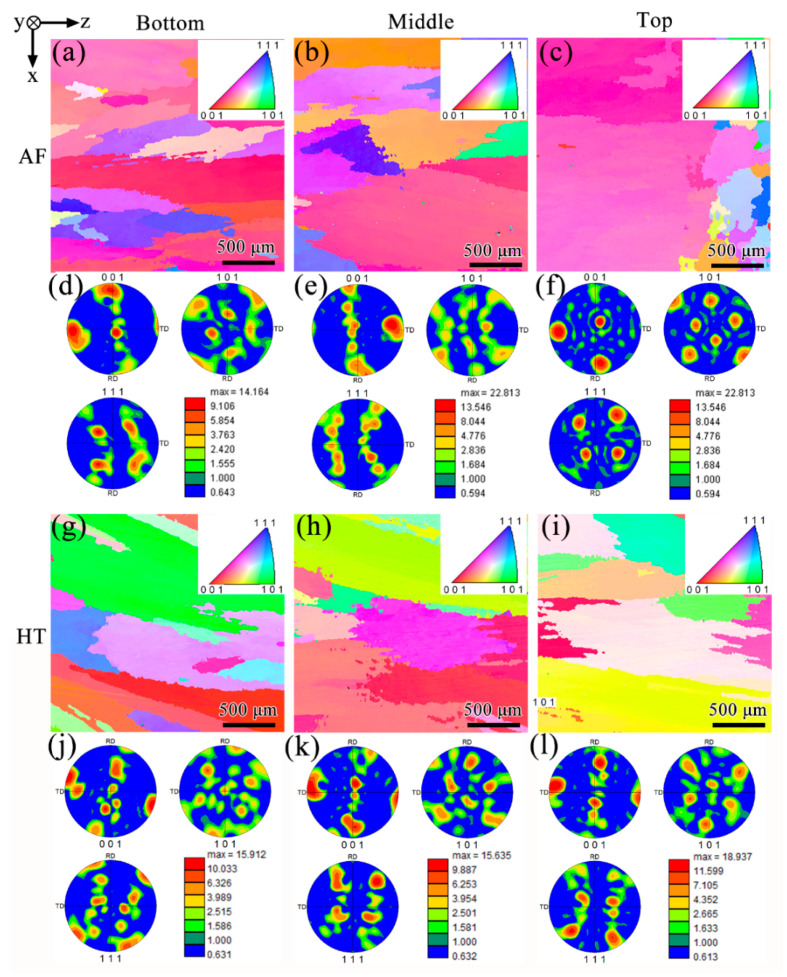
Oxford electron backscattered diffraction (EBSD) results containing inverse pole figure (IPF) of (**a**–**c**) AF samples and (**g**–**i**) HT samples, and corresponding pole figure (PF) of (**d**–**f**) AF samples and (**j**–**l**) HT samples.

**Figure 6:**
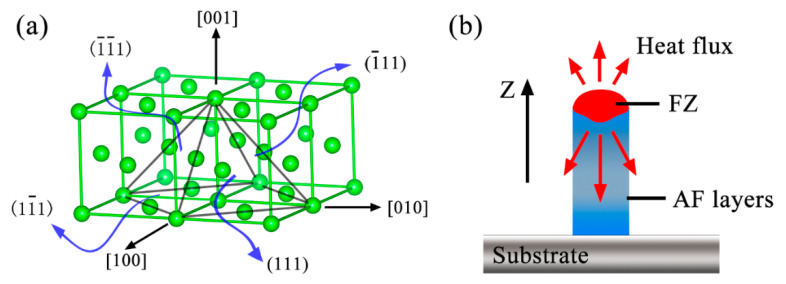
Schematics of (**a**) a pyramid made of four close-packed planes and (**b**) heat dissipation during PPAAM.

Grain orientation and growth in AF and HT samples are investigated in detail by EBSD. Figure 5a–c show the inverse pole figure (IPF) of the bottom, middle and top region of the AF samples, respectively, and the corresponding pole figure (PF) is present in Figure 5d–f. An obvious texture concentrating around <001> can be observed at different regions in AF samples. However, equiaxial grains with a disordered orientation appear in the top section. During solidification, grains are always expected to grow along the direction of heat conduction. Since the heat dissipation is the fastest in the building direction during PPAAM, in which the pre-deposits and substrate act as the heat sink, grains are more likely to align with the building direction. Furthermore, the preferred crystallographic orientation during solidification is another factor worth considering. It is known that grains can grow more quickly along a crystallographic direction with as many close-packed planes made concurrently as possible [29]. As for the IN738LC superalloy, the γ matrix is the FCC crystal structure and a pyramid can be made by the four close-packed planes of (111), ($\bar{1}$11), (1$\bar{1}$1) and ($\bar{1}\bar{1}$1), as can be seen in Figure 6a. Obviously, it is the <001> crystallographic direction that can produce these four close-packed planes simultaneously. However, heat flux is not always necessarily perpendicular to the substrate and deviates from the <001> direction at the edge of the fusion zone (FZ), as shown in Figure 6b. It can be inferred that grains will grow more quickly along the heat flux direction, which is just parallel to the crystallographic direction of <001>. Figure 5g–l exhibit the IPF and PF of HT samples, respectively. It is clear that, after heat treatment, the cube texture is replaced by a random grain structure, which has been proven to be attributed to recrystallization [30]. During heat treatment, residual stress accumulated at the stage of PPAAM is relieved and this process could serve as a driving force for the recrystallization.

### 3.2. X-ray Diffraction Analysis

**Figure 7:**
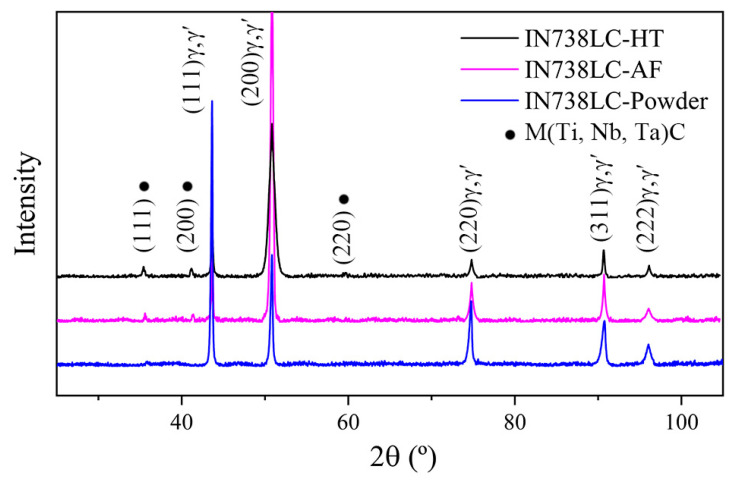
XRD results of the IN738LC powder, AF and HT samples.

The results of the XRD investigation of IN738LC powder, AF, and HT samples are summarized in Figure 7. M(Ti,Nb,Ta)C carbides are confirmed to be precipitating in both AF and HT samples. The diffraction peaks of the γ matrix emerge at 2θ = 43.7°, 50.8°, 74.8°, 90.7°, and 96.1°, which correspond to the (111), (200), (220), (311), and (222) diffraction planes of the γ matrix. One thing that should be mentioned is that it is difficult to distinguish the diffraction planes of the γ′ phase in Ni-based superalloys due to the very small difference in the lattice parameter [17]. The γ′ phase precipitates coherently from the γ matrix in the IN738LC superalloy, possessing a similar crystalline structure to the γ matrix. Additionally, the peak intensity ratios for different diffraction planes by using (I/I_max_)(hkl) were calculated and are summarized in Table 3. The IN738LC powder used in this study shows a strong (111) peak, while the AF sample exhibits a very strong (200) peak, which confirms the presence of a preferred growth of crystals during PPAAM of IN738LC superalloys. After heat treatment, the peak intensity ratio for the (111) peak increases, indicating that the preferred orientation has been weakened. This is consistent with the former EBSD result.

### 3.3. Precipitates in the AF Sample

#### 3.3.1. The γ′ Phase and Carbides in the AF Sample

**Figure 8:**
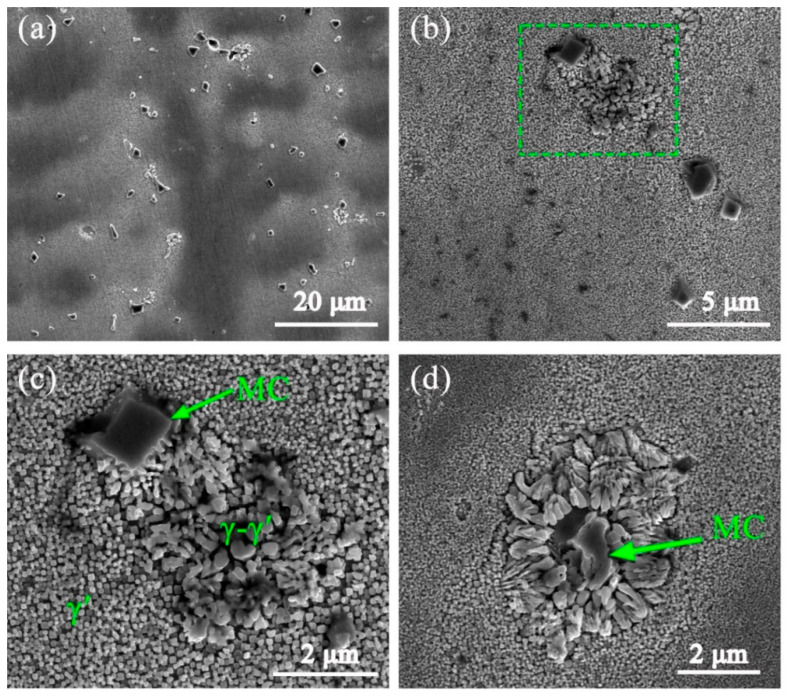
SEM micrograph of the AF sample, containing (**a**) macro morphology of precipitates, (**b**) microstructure at high magnification, (**c**) high-resolution image of the green rectangle region, and (**d**) another morphology of eutectic precipitates with MC carbides in the central point.

**Figure 9:**
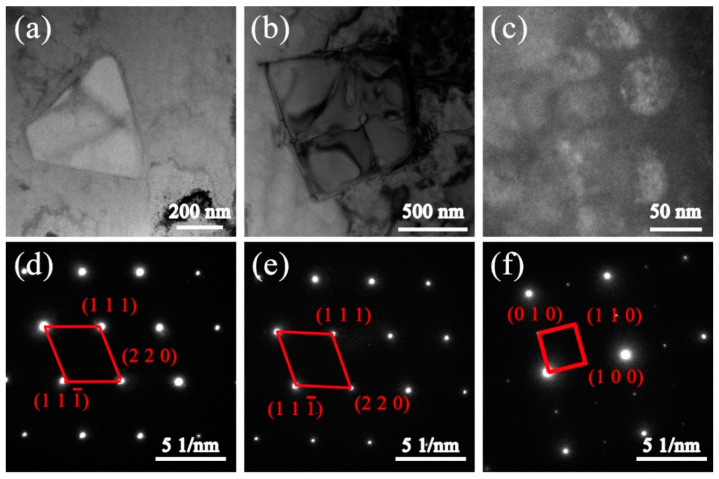
TEM micrograph of the (**a**) TiC, (**b**) (Nb,Ta)C, and (**c**) γ′ particles in the AF sample, and (**d**–**f**) corresponding selected area electron diffraction patterns (SADP), respectively.

Figure 8 shows the microstructure of AF samples; it can be seen that many precipitates, exhibiting block, particle, and irregular shapes, exist in the interdendritic region, as shown in Figure 8a. With the help of EDS and the published literature [21,31], we can be sure that the microstructure of AF samples mainly consists of MC carbides rich in Nb, Ti and Ta elements, fine γ′ particles, and some γ–γ′ eutectic constituents with irregular shapes. The EDS results of these precipitates are summarized in Table 4. Figure 8b shows the distribution of fine γ′ phases and block MC carbides. MC carbides are always enriched in the root of SDA or between PDA where Nb and Ti elements experience strong segregation, while spherical γ′ particles present a homogeneous distribution in the interdendritic region. Figure 8c shows the high-magnification SEM images of green rectangle region in Figure 8b. It can be seen that a few eutectic γ–γ′ phases with complex morphologies precipitate near MC carbides or around MC carbides, as shown in Figure 8d. TEM investigation identified the precipitates in detail. An analysis of the selected area electron diffraction patterns (SADP) in Figure 9d,e confirms that MC carbides in the AF sample include TiC with a lattice parameter of 4.32 Å, and (Nb, Ta)C with a lattice parameter of 4.45 Å. Both of these two types of MC carbides possess a cubic FCC crystal structure. Figure 9f shows the typical superlattice of the γ′ phase from the [001] zone axis, which confirms the ordered, coherent precipitation of spherical γ′ particles. The precipitation of the γ′ phase has a strong relationship with diffusion and temperature. Chen et al. have reported that there is no γ′ phase forming in the as-consolidated IN738LC samples through laser consolidation technology because of the rapid cooling rate [19]. However, bimodal γ′ phases are confirmed to be precipitating in the as-deposited IN738LC sample with the bigger particle size, ranging from 314 to 651 nm, and the fine particle size, ranging from 42 to 101 nm, through laser metal deposition technology [17]. In the present study, only fine spherical γ′ particles with a mean size of 81 nm precipitate in the AF sample and, based on area fraction analysis, the volume fraction of γ′ particles is estimated to be 35%.

#### 3.3.2. Elemental Partitioning and Phase Formation during Solidification

In the IN738LC superalloy, the elements Co, Cr, Mo, W, and Ta are generally added in the γ matrix to play an important role in the solid solution strengthening, and elements Cr, Mo, Ti and Nb can also achieve the solid solution strengthening of the γ′ phases. The distribution of various alloying elements can not only significantly affect the microstructure, but also have a strong impact on the properties. Solute redistribution during weld pool solidification is an important phenomenon which should be researched to analyze the elemental distribution. According to the solute redistribution model established by Bower et al. [32], the solute concentration in the side of a solid at the front of a solid–liquid interface can be acquired by

(2) $$C_{s}=kC_{0}{\left[1-\left(1-2\alpha k\right)f_{s}\right]}^{\left(k-1\right)/\left(1-2\alpha k\right)}$$

(3) $$\alpha =D_{s}t_{f}/L^{2}$$

where $C_{s}$ is the solute concentration, *k* is the equilibrium distribution coefficient, $C_{0}$ is the actual solute concentration, α is a diffusion parameter, $f_{s}$ is the fraction of the solid during solidification, $D_{s}$ is the solute diffusivity in the solid, $t_{f}$ is the solidification time, and *L* is the diffusion distance. Considering a constant solute diffusivity and negligible solid-state diffusion, the term α << 1 and, consequently, the above equation can be simplified as the well-known Scheil equation [33]

(4) $$C_{s}=kC_{0}{\left[1-f_{s}\right]}^{\left(k-1\right)}$$

In the present study, the cooling rate during PPAAM ranges from 216–571 K/s, and such a high cooling rate can undoubtedly suppress the solute back diffusion to some extent. Hence, it is appropriate to use Equation (4) to evaluate the distribution coefficients. At the initial stage of solidification, $f_{s}$ = 0, the first solid just starts to form in the liquid and its solid concentration is equal to $kC_{0}$. During solidification in the PPAAM, the first solid formed from liquid must be the dendritic core, so the distribution coefficient of an element can be acquired from the ratio of dendritic core composition to nominal composition. The calculated elemental partition coefficients at the initial stage of solidification are listed in Table 3. The results show that the elements W and Co with *k* > 1 can gather in the dendritic core, while the elements Nb, Mo, Ta, Ti and Zr can be more easily segregated in the interdendritic region, which is responsible for the precipitation and distribution of MC carbides.

**Figure 10:**
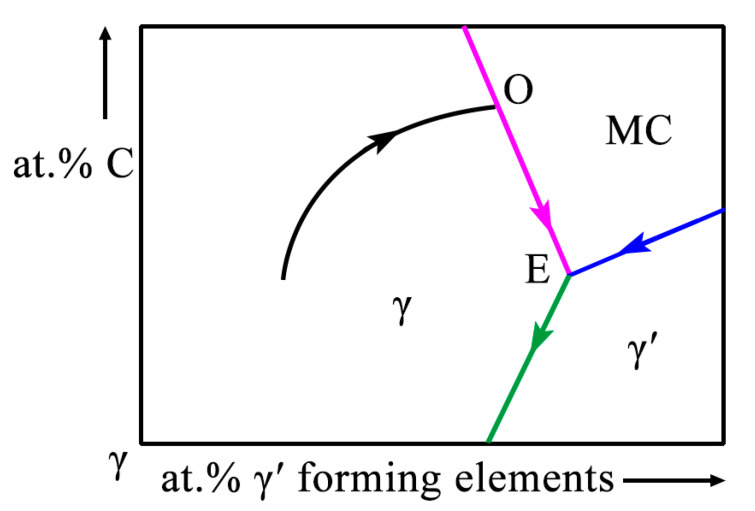
Pseudoternary diagram of γ, MC and γ′.

During solidification in the PPAAM of IN738LC, the γ matrix (dendrite) forms at the initial stage. Then, with the elements Nb, Mo, Ta and Ti segregating in the interdendritic region, MC carbides start the formation of liquid. In the next stage, the formation of γ′ begins. It is noted that elements with *k* < 1 keep on segregating during the whole solidification process. At the final stage, the enrichment of Al and Ti in the interdendritic region could lead to a eutectic reaction, L→γ + γ′, when the temperature cools down to the eutectic temperature. Another thing that should be mentioned is that MC carbides are also reported to be able to participate in eutectic reactions [22,34]. According to the pseudoternary diagram of γ, MC and γ′ in Figure 10 [22], when the enrichment of Ti and other carbides forming elements reaches point O, which represents equilibrium between the liquid, γ, and MC, the eutectic reaction, L→γ + MC, occurs. The condition of the occurrence of this eutectic reaction is the exceeded solubility limit of primary γ phases. This can also be distinguished from the morphology, location and composition of carbides, such as eutectic carbides with a coarsely lamellar morphology [34]. In the present study, no eutectic MC carbides were observed. Upon further solidification, when the chemical composition of liquid reaches equilibrium point E, the reaction L + MC→γ + γ′ can happen. Then, the remaining liquid solidifies, following L→γ + γ′. This mechanism could better account for the microstructure in Figure 8d.

### 3.4. Precipitates in the HT Sample

**Figure 11:**
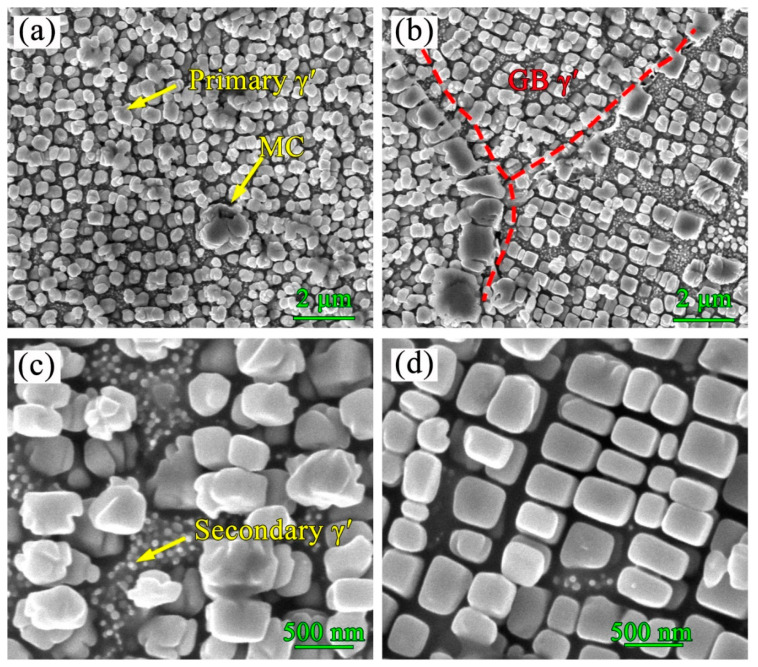
The precipitates in HT sample, (**a**) bimodal γ′ particles and block MC carbides, (**b**) grain boundary γ′ phases, (**c**) spherical γ′ particles, and (**d**) cuboidal γ′ particles.

The IN738LC superalloy is known to be metastable at high temperatures and is always accompanied by changes in the size, morphology, and distribution of γ′ phases. After standard heat treatment in the present study, IN738LC samples show a bimodal γ′ particle distribution and a dispersed distribution of MC carbides, as can be seen in Figure 11a. The coarse γ′ particles with a mean size of 385 nm account for about 42 vol.%, while the fine γ′ particles with a mean size of 42 nm account for about 25 vol.%. It is obvious that the number of γ′ phases in the HT sample increased significantly in comparison with those in the AF sample, which is attributed to the high temperature and enough aging time during heat treatment. Traditionally, the precipitation of γ′ particles in IN738LC during heat treatment experiences two forms: isothermal precipitation and continuous precipitation [19]. Coarse γ′ particles are produced according to the former pattern and this always occurs during dwelling time, while the latter pattern is beneficial to the precipitation of fine γ′ particles during a slightly slower cooling rate, such as air cooling. It is believed that the initial formation of γ′ particles is mainly affected by the first stage of heat treatment (solution, 1120 ℃/2 h), while the second heat treatment (aging, 850 ℃/24 h) actually results in the development of γ′ particles with the final morphology and size. A continuous distribution of grain boundary γ′ (GB γ′) particles can be observed in Figure 11b, which may be mainly ascribed to the dissolution of low-melting point eutectic γ–γ′, where elemental segregation of elements such as Nb, Mo, Al, and Ti is confirmed. It has been found that the concentration of these elements in an Ni-based superalloy can decrease the solidus and liquidus temperature [35]. Figure 11c,d show the different morphologies of coarse γ′ particles. Both spherical and cuboid γ′ particles are observed in the HT samples. It has been proven that the lattice mismatch between γ′ and the γ matrix plays an important role in determining the shape of γ′ phases [36,37,38]. The mismatch δ is given by $\delta =\left(\alpha_{\gamma^{\prime }}-\alpha_{\gamma }\right)/\left[\left(\alpha_{\gamma^{\prime }}+\alpha_{\gamma }\right)/2\right]$, where $\alpha_{\gamma \prime }$ is the lattice parameter of γ′ and $\alpha_{\gamma }$ is the lattice parameter of the γ matrix [39]. The coherent precipitation of γ′ phases from the γ matrix can cause coherent elastic distortion and produce a high stress field between γ′ and γ. The total energy of a solid system is usually reduced by decreasing the elastic energy. Thus, the γ′ particles with 0–0.2% δ are expected to be spherical due to their minimum interfacial energy. With the mismatch δ increasing from 0.2% to 0.5%, the morphology of γ′ particles changes from spheroid to cuboid. When the lattice misfit δ reaches a range of 0.5–1%, γ′ particles exhibit a cuboidal morphology [40,41].

During heat treatment, the γ′ phase normally evolves, following four stages: nucleation, growth, coarsening and dissolution. According to the theory of solid phase precipitation, both isothermal precipitation and continuous precipitation are mainly controlled by two vital factors: the capability of solute atom migration and the activation energy required for nucleation [42]. In Figure 11b,d, another interesting phenomenon can be observed: a lot of fine secondary γ′ particles exist between coarse γ′ particles near the grain boundary or interdendritic region, while few fine γ′ particles can be seen around cuboid γ′ particles in the dendritic core region (Figure 11d). Compared with the dendritic core region, the grain boundary or interdendritic region witness strong elemental segregation and, during the first solution heat treatment, abundant elements dissolve in the γ matrix, resulting in higher degree of supersaturation in these areas, which could reduce the activation energy required for the nucleation of γ′ particles. Hence, more fine γ′ particles can precipitate near the grain boundary or in the interdendritic region. In the subsequent dwelling time, γ′ particles grow by absorbing solute atoms from fine particles following the Ostwald ripening mechanism [43]. It can be inferred that most fine γ′ particles have been dissolved in order to guarantee the growth of cuboid γ′ particles in the dendritic core, as it is known that cuboid γ′ particles possess a greater lattice mismatch (0.5–1% δ), which can nevertheless reduce the stability of γ′ phases. During the long aging time, some cuboid γ′ particles may grow to exceed the critical size and start to change into a spherical morphology by dissolving the cuboidal corner [44]; this can be observed in Figure 11c. It is noted that γ′ particles with a cubic shape are reported, experiencing aging times up to 24 h without changing their morphology [44], and this seems to be contradicted by the results of the present study. However, fine, 35 vol.% γ′ particles were already precipitated in the AF samples during PPAAM. In fact, during the following heat treatment, this part of the γ′ particles actually experiences double “aging” treatments: one occurs at a high “solution” temperature (1120 °C) and another one takes place at a lower “aging” temperature (850 °C). This is a unique phenomenon in the PPAAM of IN738LC superalloys.

### 3.5. Cracking Mechanism in PPAAM of IN738LC

**Figure 12:**
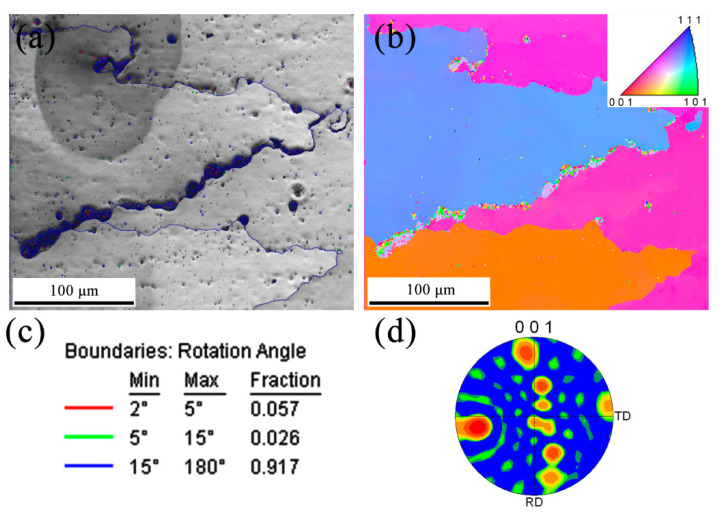
EBSD scans of the crack at the top region of AF sample, (**a**) grain boundaries colored to show different angles, (**b**) IPF image, (**c**) the color legend of grain boundaries, and (**d**) [001] pore figure of the specimen.

One common defect in most AM technologies of the IN738LC superalloy is undoubtedly micro-cracks. Figure 12 shows the EBSD results of the crack along the grain boundary at the top region of AF samples in the present study. It can be seen that cracks always exist between two oriented grains with boundaries colored in blue, which indicates that the grain boundary misorientation exceeds 15°, as shown in Figure 12a,c. As mentioned above, at the last stage of solidification, the oriented growth of grains in the PPAAM of IN738LC, could be weakened due to severe heat accumulation and a low cooling rate, as can be seen in Figure 12b,d. High-angle grain boundaries (HAGB) (misorientation > 15°) are reportedly more likely to induce the formation of cracks during the welding of Ni-based superalloys and the frequency of cracks in the as-fabricated samples will rise with the increase in the grain boundary misorientation [45,46]. This phenomenon can be explained by a theoretical model of dendritic coalescence, established by Rappaz et al. [47], during the last stage of solidification, which introduces the grain boundary energy $\gamma_{gb}$ and the solid–liquid interfacial energy $\gamma_{sl}$ to evaluate the stability of the liquid film between dendrites in the intragranular region or grains in the intergranular region. The undercooling $\Delta T_{b}$ of dendritic coalescence is given by:

(5) $$\Delta T_{b}=\frac{\Delta \Gamma_{b}}{\delta }=\frac{\gamma_{gb}-2\gamma_{sl}}{\Delta S_{f}\delta },$$

where δ is the thickness of the solid–liquid interface, $\Delta S_{f}$ is the entropy of the fusion zone, and $\Delta \Gamma_{b}$ can be seen as a parameter indicating the direction and magnitude of dendritic coalescence, which can be calculated by $\Delta \Gamma_{b}=(\gamma_{gb}-2\gamma_{sl})/\Delta S_{f}$. Given the situation in the intragranular region, including dendrites with tiny differences in their misorientation, $\gamma_{gb}$ is close to zero and, as a result, $\Delta T_{b}$ < 0. Thus, the liquid film between dendrites in the intragranular region should be unstable and dendritic coalescence would occur at the moment when two interfaces move into the distance of δ. That is why few cracks can be observed between dendrites with small misorientations in the same grains. According to the dislocation theory, the grain boundary energy can be evaluated by the Read–Shockley equation [48]:

(6) $$\gamma_{gb}=\frac{Gb\theta }{4\pi \left(1-\upsilon \right)}\left(1\;+\;\ln\frac{b}{2\pi r_{0}}-\ln\theta \right)=\frac{Gb\theta }{4\pi \left(1-\upsilon \right)}\left(1-\ln\frac{\theta }{\theta_{m}}\right),$$

where G is the shear modulus, b is the Burgers vector, θ is the grain boundary angle, υ is the Poisson ratio, $r_{0}$ is the radius of the area encompassing the energy of the dislocation core, and $\theta_{m}$ is the critical angle at which the grain boundary energy can be maximal. This equation can be used on the condition of a small grain boundary angle (<$\theta_{m}$) since $\gamma_{gb}$ can turn into a constant value (maximum $\gamma_{gb}$) when θ > $\theta_{m}$ [46]. Obviously, $\gamma_{gb}$ can increase at HAGB. When $\gamma_{gb}$ > $2\gamma_{sl}$, the dendritic coalescence undercools $\Delta T_{b}$ > 0, and as a result, the liquid film between dendritic arms is stable until a lower temperature is reached due to the “repulsive” effect between the two interfaces. It is known that thermal stress increases with decreasing temperature during solidification. Thus, the thermal stress can easily concentrate around the liquid film due to its stability under the circumstances of $\Delta T_{b}$ > 0. Then, as the thermal stress exceeds the dendrites’ resistance limit, liquid film can be consequently pulled apart and thus liquation cracks will be generated. Accordingly, the cracking susceptibility of larger grain boundary misorientation will be stronger due to the better stability of the liquid film and the thermal stress concentration.

**Figure 13:**
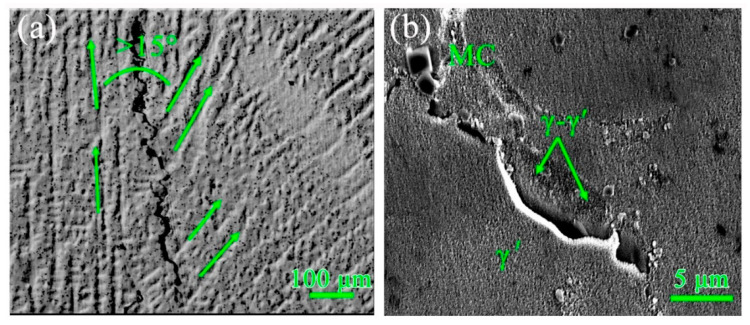
Cracks in the AF sample. (**a**) Optical microscope (OM) image of grain boundary cracks with green arrows indicating different growth directions of dendrites in two grains; (**b**) SEM image of cracks decorated by fine γ′ particles, γ–γ′ eutectics and some MC carbides.

**Figure 14:**
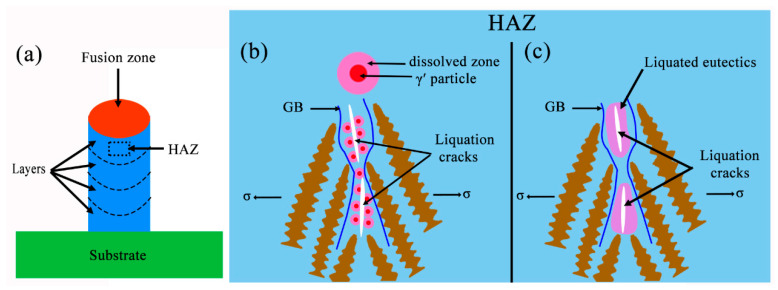
Schematic diagram of two liquation mechanisms, (**a**) the location of heat-affected zone (HAZ), (**b**) constitutional liquation of γ′ particles, and (**c**) localized melting of γ–γ′ eutectics.

Figure 13a shows a grain boundary crack along the depositing direction with a high grain boundary misorientation (>15°), in accordance with the EBSD results. A high-magnification SEM image of the crack in Figure 13b demonstrates that many fine γ′ particles, γ–γ′ eutectics and some MC carbides can be found adjacent to the crack, which may imply the occurrence of constitutional liquation of γ′ particles or the re-melting of γ–γ′ eutectics. These two liquation mechanisms are illustrated in Figure 14. The constitutional liquation mechanism was firstly interpreted by Pepe and Savage [49]. During the PPAAM process, pre-deposited layers experience actual “heat treatment” from the subsequent layers, and this re-heated region can also be regarded as the heat-affected zone (HAZ). This complex heat history can give rise to complex metallurgical reactions including melting, re-melting, partial melting and cyclic annealing. The constitutional liquation of γ′ particles was observed in the HAZ of IN738LC through Tungsten Inert Gas (TIG) welding [22]. The main requirement for the occurrence of constitutional liquation of an intermetallic compound is its existence when heating the alloy to a temperature equal to or above the equilibrium eutectic reaction temperature. Thus, the susceptibility of γ′ particles to constitutional liquation should fundamentally be related to the solid-state dissolution behavior. It has been reported that the solid-state dissolution of non-coherent precipitates is mainly diffusion governed, and constitutional liquation can take place easily, while the solid-state dissolution of coherent precipitates is typically interface controlled, and constitutional liquation can seldom occur [50]. Unfortunately, the γ′ particles lose their coherence as the size exceeds a critical dimension. In the present study, the size of γ′ particles precipitated in the AF samples is about 81 nm. Consequently, the constitutional liquation of γ′ particles is almost impossible and no evidence can confirm the occurrence of this process. Hence, the cracks in the present work are mainly attributed to the localized melting of γ–γ′ eutectics during subsequent depositions, which can be confirmed in Figure 13b. During the complex heat history in the PPAAM of IN738LC, the low-melting point γ–γ′ eutectics can be easily re-melted in the “HAZ” and can then generate liquid film along the grain boundaries. It is known that weld cracking always results from the competition between the mechanical driving force, internal stress/strain, and the material’s intrinsic resistance [51]. The liquid film along the grain boundaries due to the localized melting of γ–γ′ eutectics can induce a low ductility in the alloy and thus results in liquation cracks on the condition of enough internal strain under a complex thermal history.

### 3.6. Mechanical Performance

#### 3.6.1. Microhardness

**Figure 15:**
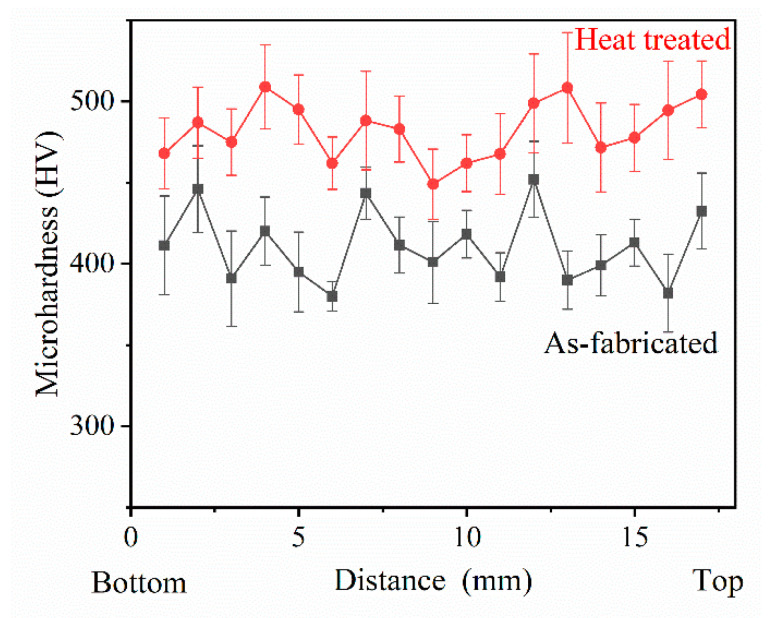
Microhardness of the AF and HT samples depending on their distance from the bottom.

Figure 15 shows the microhardness variation profiles of AF and HT samples, depending on their distance from the bottom. It is clear that the microhardness of both AF and HT samples witness an obvious fluctuation, which may be ascribed to their nonuniform microstructure. The average value of microhardness in the AF sample is about 411 HV, while that in the HT sample increases to about 483 HV. Apparently, upon heat treatment, the microhardness of the AF sample experiences a significant increase (17%). It is noted that there is no obvious regularity in the microhardness along the deposition direction, although the dendritic arm spacing and cooling rates were confirmed to be different from the bottom to the top region of the AF sample. It is well known that the high strength of IN738LC superalloys is primarily acquired from the precipitation of coherent γ′ phases. During PPAAM of IN738LC, 35% vol γ′ particles with average sizes of 81 nm precipitated in the AF sample, which may be mainly responsible for the microhardness variation in the AF sample. After heat treatment, abundant γ′ particles (~67% vol) formed during solution and aging treatment, resulting in the increase in microhardness in the HT sample.

#### 3.6.2. Tensile Properties

Tensile tests at room temperature were also carried out to investigate the mechanical performance of AF and HT samples. Ultimate tensile strength (UTS), yield strength (YS) and elongation (EL) are summarized in Table 5. The UTS and YS of IN738LC samples fabricated by PPAAM and other AM technologies, such as LMD, SLM and LSF, are remarkably higher than those of cast parts. The differentiation in the tensile strength of samples fabricated by different technologies is mainly attributed to the macro- and microstructures, such as the size and distribution of γ′ phases as well as the morphology of grains. Traditionally, the primary strengthening mechanism of γ′ phases is the coherent relation with the γ matrix. However, as mentioned above, when the size of γ′ phases exceeds a critical dimension, this coherent relation is devastated. The as-fabricated samples with a bimodal distribution of γ′ particles fabricated by LMD show a higher tensile strength than the HT samples, which results from the coarsening of γ′ particles [17]. Xu et al. ascribed the higher YS of as-fabricated samples to fine grains and more grain boundaries because the grains in as-fabricated samples are no more than 200 μm, while the grains in cast parts are always on the millimeter scale, and the grain boundaries are able to hinder dislocation motion [20]. In the present study, fine γ′ particles play an important role in the higher tensile strength in comparison with cast parts and, after heat treatment, more precipitation of bimodal γ′ particles with moderate sizes contributes to the excellent tensile strength of HT samples. It can be inferred that the mechanical performance of IN738LC samples fabricated by PPAAM in this work still has the potential to be enhanced through adjusting the heat treatment to acquire a more appropriate size and distribution of the γ′ phases.

## 4. Summary and Conclusions

In the present study, an IN738LC thin wall free from macro-cracks is successfully fabricated by PPAAM, the microstructure and mechanical performance of AF and HT samples are investigated in detail and the primary conclusions can be summarized as follows.

1. The AF samples exhibit epitaxially growing columnar dendrites along the building direction with different SDA spacing. Because of the heat accumulation during PPAAM, the SDA spacing of the bottom, middle, and top region is found to be 12.1 μm, 16.2 μm, and 21.5 μm, respectively. The grain morphology also changes from columnar dendrites to cellular dendrites and equiaxial dendrites due to the variational thermal gradient (G) and grain growth rate (R), which can result in constitutional undercooling.
2. The precipitates of AF samples mainly consist of MC carbides rich in Nb, Ti and Ta, fine spherical γ′ particles with an average size of 81 nm, accounting for about 35 vol.%, and some γ–γ′ eutectics with irregular shapes. Elements with a partition coefficient *k* < 1, such as Nb, Mo, Ta, and Ti, are easy to segregate in the interdendritic region, which could lead to a eutectic reaction, L→γ + γ′, at the final stage of solidification. Moreover, some MC carbides are confirmed in the reaction L + MC→γ + γ′.
3. After standard heat treatment, the HT sample shows a bimodal γ′ particle distribution. The coarse γ′ particles with a mean size of 385 nm account for about 42 vol.%, while the fine γ′ particles with a mean size of 42 nm account for about 25 vol.%. Both spherical and cuboidal γ′ particles are observed and nonuniformly distributed due to the elemental segregation. The morphology evolution is ascribed to the virtually double “aging” treatments.
4. Crack formation tends to occur along high-angle grain boundaries (HAGB) with the misorientation exceeding 15°, at which point dendritic coalescence undercooling occurs $\Delta T_{b}$ > 0 and results in the better stability of the liquid film and thermal stress concentration. In addition, the localized melting of γ–γ′ eutectics can also induce a low ductility in the alloy and thus results in liquation cracking on the condition of enough internal strain under a complex thermal history.
5. Both AF and HT samples exhibit higher UTS and YS, but slightly lower ductility compared with cast parts. A further study, adjusting the heat treatment to acquire a more appropriate size and distribution of γ′ phases, may further improve the mechanical performance of IN738LC samples fabricated by PPAAM.

## Figures and Tables

**Table 1:** The chemical composition of IN738LC powder (wt.%).

Elements	C	Co	Cr	Mo	W	Nb	Ta	Al	Ti	Zr	B	Ni
wt.%	0.11	8.5	16.0	1.75	2.60	0.9	1.75	3.40	3.40	0.05	0.01	Bal.

**Table 2:** Main process parameters of PPAAM.

Layers	Parameters		Average Power P/W
	Peak Current I/A	Voltage U/V	
1	200	24.7	3952
2	195	24.4	3806
3	190	24.3	3694
4	185	23.8	3522
5	180	23.5	3384
6	175	23.2	3248
7–8	170	22.5	3060
9–10	165	22.4	2956
11–12	160	22.0	2816

**Table 3:** Peak intensity variation in XRD examination.

Samples	Peak Intensity Ratio (%) [(I/I_max_)(*hkl*)]				
	(111)	(200)	(220)	(311)	(222)
Powder	100	41	24	17	7
As-fabricated	16	100	8	11	3
Heat treated	33	100	10	18	8

**Table 4:** Chemical composition of precipitates in the AF sample by EDS.

Elements	Ni	Co	Cr	Mo	W	Nb	Ta	Al	Ti	Zr	C
Dendritic core	63.98	9.18	16.16	1.66	3.15	0.432	1.19	3.21	2.21	0.008	-
*k*	1.04	1.08	1.01	0.85	1.21	0.48	0.68	0.94	0.65	0.16	-
MC (at, %)	6.46	1.14	3.24	1.7	1.91	6.36	8.67	-	20.64	-	49.88
γ′ (at, %)	60.28	6.54	12.56	1.25	1.43	0.68	0.54	11.64	5.08	-	-
Eutectic γ–γ′ (at, %)	61.38	7.78	14.43	1.16	1.34	0.69	0.75	8.46	4.01	-	-

**Table 5:** Tensile properties of the IN738LC components fabricated by PPAAM vs. other manufacturing methods.

Manufacturing Method	Condition	UTS (MPa)	0.2%YS (MPa)	%EL
PPAAM	As-fabricated HT	1084 ± 43 1272 ± 58	902 ± 36 1081 ± 56	6.0 ± 0.9 3.5 ± 0.4
LMD [17]	As-deposited	1392	1350	1.13
	HT	1117	1038	2.76
SLM [52]	As-SLMed	1162–1184	786–933	8.4–11.2
LSF [20]	As-deposited	1074	871	10.8
LMD [53]	As-deposited	1093–1116	1058–1073	1.2–1.4
Cast [52]	-	945	765	7.5
